# Using Game Theory to Understand Systemic Acquired Resistance as a Bet-Hedging Option for Increasing Fitness When Disease Is Uncertain

**DOI:** 10.3390/plants8070219

**Published:** 2019-07-12

**Authors:** Gregory J. Reynolds, Thomas R. Gordon, Neil McRoberts

**Affiliations:** 1 Forest Health Protection, U.S. Forest Service, 333 Broadway Blvd. SE, Albuquerque, NM 87102, USA; 2 Department of Plant Pathology, University of California, One Shields Avenue, Davis, CA 95616, USA

**Keywords:** systemic acquired resistance, induced resistance, game theory, plant pathogens, evolution

## Abstract

Systemic acquired resistance (SAR) is a mechanism through which plants may respond to initial challenge by a pathogen through activation of inducible defense responses, thereby increasing resistance to subsequent infection attempts. Fitness costs are assumed to be incurred by plants induced for SAR, and several studies have attempted to quantify these costs. We developed a mathematical model, motivated by game-theoretic concepts, to simulate competition between hypothetical plant populations with and without SAR to examine conditions under which the phenomenon of SAR may have evolved. Data were gathered from various studies on fitness costs of induced resistance on life history traits in different plant hosts and scaled as a proportion of the values in control cohorts in each study (i.e., healthy plants unprimed for SAR). With unprimed healthy control plants set to a fitness value of 1, primed healthy plants incurred a fitness cost of about 10.4% (0.896, *n* = 157), primed diseased plants incurred a fitness cost of about 15.5% (0.845, *n* = 54), and unprimed diseased plants incurred a fitness cost of about 28.9% (0.711, *n* = 69). Starting from a small proportion of the population (0.5%) and competing against a population with constitutive defenses alone in stochastic simulations, the SAR phenotype almost always dominated the population after 1000 generations when the probability of disease was greater than or equal to 0.5 regardless of the probability for priming errors.

## 1. Introduction

Over the course of their evolution, plants have developed defenses to reduce damage by pathogens. In addition to constitutive defenses, such as cell walls or bark, plants may also resist attack by these parasites with inducible defenses. Systemic acquired resistance (SAR) is a mechanism by which plants may respond to an initial pathogen or microbe challenge by activating some of these inducible defense responses, thereby increasing resistance to subsequent infections [1]. The term “priming” is used to refer to the set of molecular, biochemical, and physiological changes that occur in a plant when an environmental cue indicates an increased probability of pathogen attack. It is important to note the distinction between priming of active resistance mechanisms and active resistance itself. Although SAR depends on priming, the two phenomena, priming of resistance responses and induction of SAR, are not the same [1].

The question of whether priming itself has an appreciable fitness cost in the absence of disease is a long-standing topic of discussion [2] and is one of the issues we wished to explore from a theoretical perspective. Because effective SAR depends on an effective priming mechanism, we consider the potential fitness costs or benefits of priming and SAR as components of overall fitness costs (or benefits) of SAR in comparison with resistance based entirely on constitutive (i.e., not inducible) components.

Evidence for SAR has been demonstrated in all angiosperms investigated to date [3], as well as in the conifer Monterey pine (*Pinus radiata* D. Don) when infected with the pitch canker pathogen, *Fusarium circinatum* Nirenberg & O’Donnell [4]. Fitness costs due to SAR are believed to occur because of reallocation of resources to associated defenses, which may include production of the signaling molecule salicylic acid [5], cell wall material [6], phytoalexins, and pathogenesis-related proteins [7]. Salicylic acid appears to be involved in priming for SAR, though the specific mechanism involved remains unknown. The topic of fitness costs and/or benefits associated with induction of SAR has been the subject of several recent reviews [8,9,10] that have focused largely on experimental studies of the mechanisms of SAR and potential effects on plant fitness components. In contrast, relatively few attempts have been made to study SAR from a theoretical and quantitative perspective, and existing reports [11,12] generally focus on an ecological perspective and do not directly connect with the body of literature in experimental plant physiology. Similarly, with few exceptions [13], the bulk of the plant physiology and genetics literature does not reference theoretical modeling on the subject.

In this paper, we connect theoretical and experimental approaches to studying SAR using a simple game theory approach. We used a synopsis of previously published results to estimate cost parameters for SAR priming and a linear expected cost model to examine the consequences for relative fitness of hypothetical SAR and non-SAR phenotypes in a stochastic environment in which disease is uncertain.

Game theory [14,15] has previously been applied to both plant ecology [16] and phytopathology [17]. McRoberts et al. [18] applied game-theoretical approaches in developing a framework for analyzing disease risk and rational decision making for disease control in stochastic environments. In its original formulation, game theory referred to the mathematical study of choice problems when individuals’ choices directly affected gains and losses of any suitable context-dependent variable, the classic example being the “prisoners’ dilemma” game [15]. However, the term “game theory” is now widely applied to optimization problems in which selection of options in the face of uncertainty is required to maximize (or minimize) a quantity of interest. Of particular relevance in the current context, there is a long history in plant ecology of referring to the problem of optimizing fitness in stochastic environments as a type of game; one in which the individual players play against Nature. In this type of game, the “choices” of individuals do not directly affect each other’s payoffs but do so only indirectly after the outcome of each separate game against Nature is decided. The paradigm example of this concept is Ellner’s theoretical analysis of seed germination in desert annual plants [19].

The concept of “games against Nature” is also seen in other contexts, for example in information theory [20] and model selection and inference [21]. Donaldson-Matasci et al. [22] provide a link between information-theoretic, game-theoretic and ecological studies of fitness by examining the fitness costs of bet-hedging strategies in games against Nature in information quantities, providing a way to quantify the fitness value of the information contained in environmental cues. The error-rates associated with responding to environmental cues with alternative phenotypes that have differential fitness values in different types of environment can be expressed in terms of the likelihood ratios associated with the response; the likelihood ratios being information quantities [23,24].

Here, we use a similar approach to examine the interaction between environmental uncertainty (i.e., the likelihood of disease in a given fixed duration generation), the accuracy of the SAR priming mechanism, and the fitness cost of faulty priming on the relative fitness of hypothetical plant phenotypes that either have SAR-based resistance or lack SAR, instead relying on constitutive resistance alone. The choice of analytical approach was prompted by the need to measure the fitness cost of priming in both the presence and absence of disease [2]. We derive an expression for expected fitness as a probability-weighted average of the fitness values when disease is present or absent. Over the long term in the simulations, the average per-generation fitness for any phenotype is the geometric mean of fitness values obtained in each generation.

The formulation of expected fitness in the model as a linear function of disease probability allows us to easily to calculate a threshold probability for disease at which greater relative fitness switches between SAR and constitutive (CON) phenotypes. We show that, within the framework we use, the threshold value of long term disease incidence at which higher fitness switches from one phenotype to another depends on the following: the fitness of each phenotype when disease is absent, the fitness of each phenotype when disease occurs, the accuracy of the priming mechanism in differentiating environments in which disease will occur from those in which it will not, and the probability of the environment being one in which disease occurs.

We examine two variations on this theme, one in which SAR occurs as an additional form of resistance which complements constitutive resistance (i.e., the SAR population has both constitutive resistance and the capability for induced resistance) and a second situation in which we assume SAR and constitutive resistance are alternative strategies (i.e., the SAR population does not have constitutive defenses). The synoptic analysis of previous experimental results of fitness costs allows us to estimate a threshold value for the probability of disease based on experimental observations. We then examine the consequences for changing the accuracy of the priming mechanism on the relative fitness of SAR when the probability of disease is close to and distant from this threshold value. This allows us to make inferences about the conditions under which priming of SAR first evolved. Our study is the first attempt we are aware of to link experimental estimates of fitness costs and benefits of SAR to a theoretical model for selection of SAR through competition. A brief report on the concept has been published as a conference abstract [25].

## 2. Results

### 2.1. Synoptic Analysis

Seven studies were selected to develop the model based on the quality of the data and nature of the work, representing a total of 15 experiments. Compiled studies focused on a range of plant hosts and inducers of resistance, including Monterey pine induced by *Fusarium circinatum* [26], wheat (*Triticum aestivum* L.) induced chemically [27], tobacco (*Nicotiana tabacum* L.) induced by *Peronospora hyoscyami* (Rabenh.) de Bary [28], barley (*Hordeum vulgare* L.) induced chemically [29] and by an avirulent *Blumeria graminis* (DC.) Speer [30], and *Arabidopsis thaliana* (L.) Heynh. induced chemically [31,32]. Because of potential pleiotropic effects of mutations, studies showing fitness costs or benefits in mutant plant lines under- or over-expressing SAR-related genes were omitted from the analysis [13,33]. Three studies allowed for calculation of all four variables [26,29,32]. One study allowed for calculation of all variables but primed diseased plants [30]. The remaining three studies allowed for calculation of variables for only unprimed and primed healthy plants.

**Figure 1:**
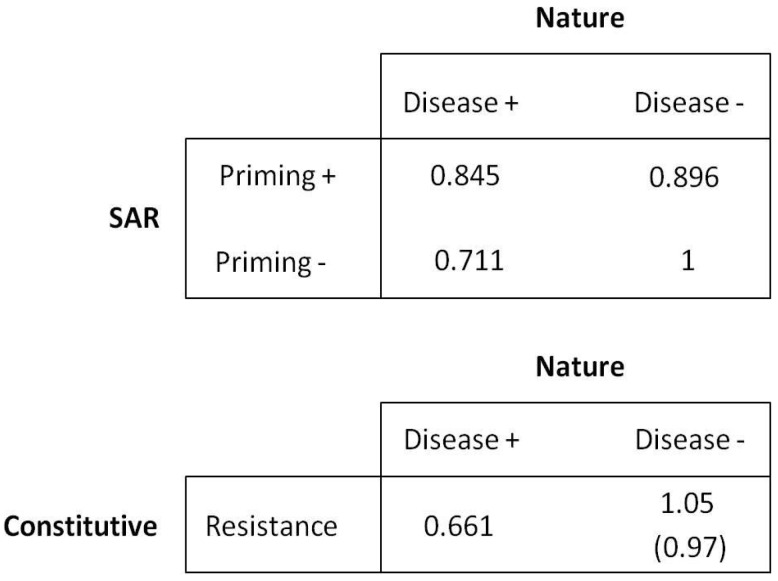
Payoff matrices showing fitness values for two two-player games versus Nature involving hypothetical populations of plants under two scenarios of systemic acquired resistance (SAR) and constitutive resistance (CON) strategies. SAR competes against Nature in the first game using one of two strategies (priming + or priming -), while CON competes against Nature using only constitutive resistance as a strategy. In one scenario strategies are complementary, and the SAR population has constitutive resistance as well as the capability for induced resistance. In the alternative scenario (parentheses), one population has SAR alone and one population has CON alone. The populations may be either primed for resistance or unprimed, and they may be either healthy or subjected to a disease epidemic. The constitutive population is unaffected by priming.

A summary of the experiments used to calculate parameters for the model is shown in Table 1. Primed healthy plants tended to incur a growth cost of about 10.4% (0.896, *n* = 157). Unprimed diseased plants incurred a growth cost of about 28.9% (0.711, *n* = 69), and primed diseased plants 15.5% (0.845, *n* = 54). Fitness for the constitutive population in the absence of disease was assumed to have a 5% benefit (1.05) over the SAR population when resistance strategies were complementary, and fitness for the constitutive population in the presence of disease was assumed to have an additional 5% cost (0.661) compared to the unprimed diseased population. In the case where constitutive and SAR types are alternatives, the constitutive type was assumed to suffer a 3% fitness cost (0.97) compared with the SAR type in the absence of disease. These costs and benefits are incorporated into a payoff matrix which determines the basic structure of the game theory model (Figure 1).

### 2.2. Numerical Simulation

**Figure 2:**
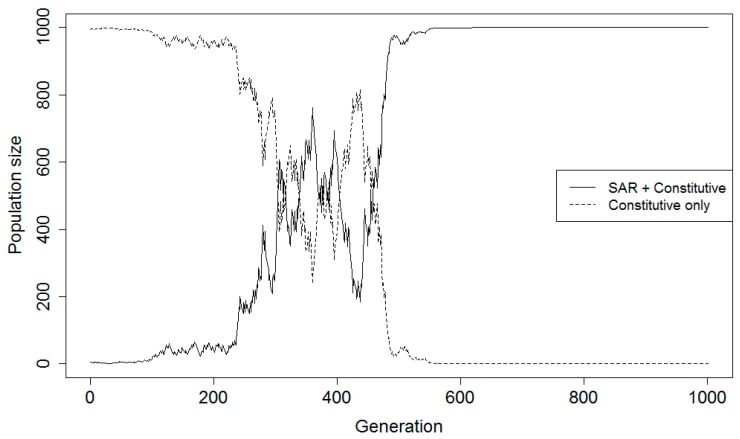
Example projections for one run of a game theory model simulating hypothetical plant populations with (SAR + Constitutive) or without (Constitutive only) the capability for systemic acquired resistance (complementary resistance strategies) over 1000 generations when disease certainty was 40%, the probability for a priming false positive was 40% and the probability for a false negative was 20%.

**Figure 3:**
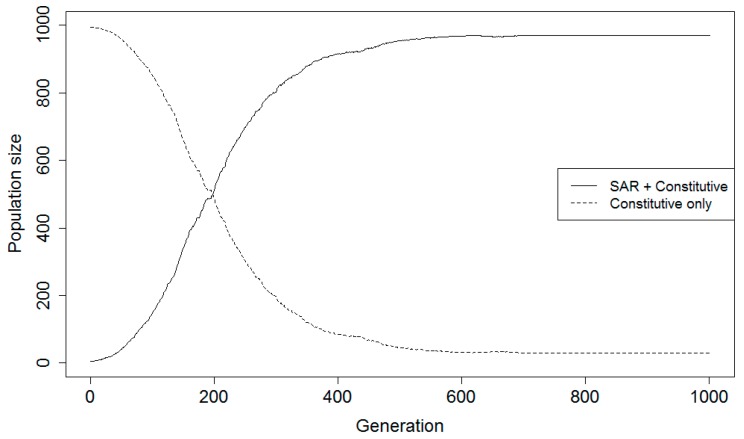
Example projections for averages of 100 simulation runs of a game theory model simulating hypothetical plant populations with (SAR + Constitutive) or without (Constitutive only) the capability for systemic acquired resistance (complementary resistance strategies) over 1000 generations when disease certainty was 40%, the probability for a priming false positive was 40%, and the probability for a false negative was 20%.

Population projections for a representative individual simulation run across at least 1000 generations for a sample parameter value combination are shown in Figure 2. Averaged projections across 100 simulation runs for the same sample parameter value combination are shown in Figure 3. A summary of the various parameter value combinations was generated for complementary (Table 2) and alternative (Table 3) strategy models listing the average time specific events took to occur. For each parameter value combination, the average generation in which the SAR population transitioned to dominate the population (>50%) is identified as the transition generation, if such a transition occurred. The average generation where the proportion of the population consisting of the SAR strategy stabilized is referred to as the plateau; this is the generation by which time the SAR population had either completely eliminated the constitutive population or had been completely eliminated itself. The average final population size for the SAR population is also noted.

**Figure 4:**
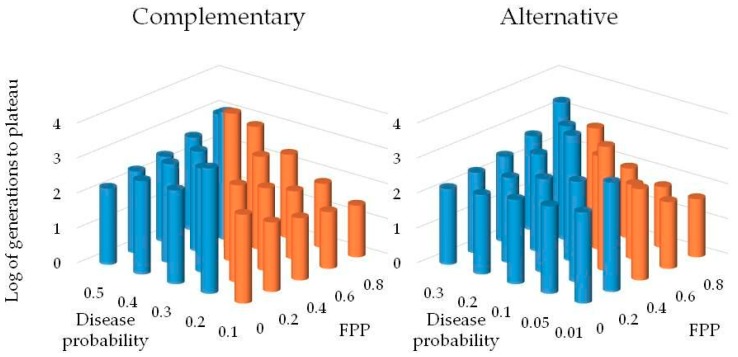
Log of generations that occurred before population plateau in our game theory model for each combination of disease probability and false positive probability where FPP is the probability of false positive priming. The systemic acquired resistance population dominated in blue entries, and the constitutive population dominated in red entries. Note that the scales for disease probability differ between alternative (SAR population does not have constitutive defenses) and complementary (SAR population has both constitutive defenses and the potential for induced resistance) strategy models.

For the scenario where SAR and CON are complementary, the SAR population was always eliminated when disease certainty was 10%, regardless of the priming error probabilities (Table 2). The SAR population had higher fitness than the constitutive population in 87% of cases at a disease certainty of 20% when priming was assumed to be perfectly accurate (Figure 4). The SAR population had higher fitness than the constitutive population in at least 98% of cases at a disease certainty of 30% when error rates were ≤ 40% for false positive priming (FPP) and ≤ 20% for false negative priming (FNP). At disease certainty greater than or equal to 40%, the SAR population had higher fitness than the constitutive population on average except for at the highest error rates, dominating at least 66% of simulation runs at all other error rates. At disease certainty greater than or equal to 50%, the SAR population dominated at least 89% of the time even with the highest priming error probabilities.

For the scenario where SAR and CON are alternative strategies, the SAR population had higher fitness than the CON population when disease incidence was 1–5% at error rates FPP ≤ 20% and FNP ≤ 10% and was always eliminated at higher error rates (Table 3). The SAR population had higher fitness than the CON population when disease incidence was 10% at error rates FPP ≤ 40% and FNP ≤ 20% and dominated in at least 99% of cases, while again always being eliminated at higher error rates (Figure 4). When disease incidence was 20%, the SAR population dominated simulation runs at error rates FPP ≤ 60% and FNP ≤ 30% in at least 97% of cases and was eliminated at error rates FPP = 80% and FNP = 40%. At disease incidence greater than or equal to 50%, the SAR population dominated regardless of error rates in at least 96% of cases.

## 3. Discussion

The scenario in which SAR complements constitutive resistance is the more realistic of the two scenarios examined. At relatively low values for disease incidence and/or under highly inefficient disease prediction by priming, the constitutive population was able to resist invasion by the SAR type consistently across 100 replicate simulations. At disease certainty greater than or equal to 50%, the SAR population dominated on average regardless of error rates. The results suggest that even with relatively inefficient priming accuracy, SAR provides enough of a selective advantage, under the combination of measured and assumed fitness costs, across a broad range of moderate to high disease probabilities, indicating that it is likely to become established. For the scenario where SAR and CON are alternative strategies, the CON population was able to resist invasion by the SAR type only for moderate to high error rates even when disease incidence was 1%. Under the alternative strategy scenario, the SAR strategy is more fit in most cases and would likely have been regardless of long- term disease pressure.

In spite of its essential simplicity, the model used here produces results that are in agreement with previous theoretical analyses of the evolution of inducible resistance. For example, using an extended Lotka–Volterra framework, Frank [12] found that under reasonable assumptions about the impact of disease on host life-history parameters, inducible resistance was likely to dominate as pathogen abundance increased. He also found that the transition between “preference” for induced and non-induced states was relatively sharp. Both of these findings are mirrored in the results we obtained in this study.

The expected fitness calculations make it apparent how resistance priming acts as a bet-hedging strategy. Recall that, in the model, environments either do or do not confront the plant with disease. The values of E(*W*), calculated as probability weighted mixtures of the *W* obtained in the presence and absence of disease, can be thought of as the long-term fitness of each strategy over a large number of generations. Consider the scenario where SAR evolves as an additional resistance strategy to complement constitutive resistance. When disease is absent, priming reduces fitness in comparison with CON, but increases it when disease is present (*p* = 1). In effect, some of the potential fitness available to CON when *p* = 0 is forgone to “buy” increased fitness (compared with CON) when *p* = 1. The SAR strategy hedges between the *W* values obtained by the CON strategy and produces a higher fitness payoff as long as the probability of disease is greater than p*.

Only three of the seven studies utilized for the synoptic analysis contained data for all four parameters, with the others only assessing primed and unprimed plants in the absence of disease. The true costs and/or benefits associated with SAR cannot be accurately described in the absence of a pathogen challenge. In order to utilize as much data as possible for our model, we used studies that did not include all four parameters even though this resulted in estimates for each parameter being generated from varying sample sizes. Transferring experimental estimates of fitness costs to a theoretical model for examining possible evolutionary dynamics highlights the necessity of estimating fitness both in the presence and absence of pathogen challenge [2]. Future studies assessing the impacts of SAR on fitness, however, should examine both healthy and pathogen-challenged populations to more accurately describe costs and/or benefits associated with the mechanism.

While our model averages across levels of resistance induction and other factors from the original studies, the actual impact of SAR varies with these factors depending on specific conditions present in the system [27,31,32]. Thus, the SAR population could theoretically dominate more rapidly under environmental conditions favorable to this phenotype, and the constitutive population could likewise survive for longer periods of time in environments less favorable to the SAR strategy. Regardless, our simulation results indicate that using parameters estimated from experiments combined with reasonable assumptions for unmeasured parameters, SAR is likely to be favored in environments where disease is moderately to highly probable. The ubiquity of the mechanism throughout all investigated plant species is consistent with this expectation [3].

Rates for false positive and false negative priming are related, with FPP always being twice the magnitude of FNP, to simplify the model. Both error rates, however, can also theoretically be influenced by similar factors. For example, a false positive could occur when environmental conditions early in an individual generation are favorable for pathogen infection, leading to priming for SAR, but are not sustained long enough into the season for the development of an epidemic [33]. Similarly, extended favorable conditions occurring later in an individual growing season in the absence of an early priming event could lead to a false negative. True false negatives would be less likely to occur, however, because even a rapid epidemic would induce SAR responses in plants not killed outright. Furthermore, because of interactions with avirulent microbes, plants growing in the field are likely always induced for SAR to some degree [34,35]. In the absence of a direct priming event, there could be a slower defense response when disease epidemics occur, leading to the increased fitness cost within the model, but defining false negatives as always half as likely as false positives makes intuitive sense.

For the complementary strategy model, we assumed a 5% benefit for healthy plants from the constitutive population compared to the healthy unprimed plants from the SAR population because of a presumed cost of having the capability for SAR even in an unprimed state [36]. Though some experiments using mutants deficient in genes required for SAR showed no significant differences in the absence of pathogen challenge [13,33], actually quantifying any benefit experimentally would be virtually impossible because of the number of genes that would need to be knocked out as well as potential pleiotropic effects of mutation. Our assumption that diseased plants from the constitutive population experience an additional 5% fitness cost compared with unprimed diseased plants in the SAR population, because of induction of resistance once disease occurs even in the absence of priming, is similarly difficult to assess experimentally.

Overall, the SAR population tended to dominate the CON for both alternative and complementary strategy scenarios and regardless of priming error probability when the probability for an epidemic was over 50%. Even at lower disease levels, the SAR population generally had higher fitness when priming error probabilities were moderate to low. Given the mechanism’s ubiquity across all investigated plant species [3], it is not surprising that SAR would consistently have higher fitness across a wide range of disease conditions. Furthermore, the fact that SAR is active against various pathogens and not just the inducing agent [37] suggests that priming error probabilities may not significantly reduce the fitness of the plant host, as a false positive priming error for one pathogen would not necessarily be costly if a different pathogen is prevalent.

## 4. Materials and Methods

### 4.1. Synoptic Analysis of Experimental Data

Data were compiled from various studies on fitness costs associated with induced resistance to plant pathogens. Seed yield data were collected when available as a direct measurement of fecundity and fitness; growth rate data were used when yield data were missing as a proxy for fitness. In studies with multiple treatments (i.e., varying nutrient regimes or levels of resistance induction), each treatment was considered as a separate experiment. Four variables were calculated from each experiment when possible, representing relative fitness for unprimed healthy plants, primed healthy plants, unprimed diseased plants, and primed diseased plants. The seed yield values for unprimed healthy plants within each experiment were set equal to 1 to standardize across all experiments. Fitness for the three remaining treatments was calculated as a proportion of the standardized unprimed healthy controls. Effects from each experiment were scaled by multiplying measured fitness values by that experiment’s sample size and averaging across all experiments by dividing by the combined sample size of all utilized studies.

### 4.2. Game Theory Model

The basic structure of the game is described in the payoff matrix (Figure 1). Following notation used commonly in population genetics, we used *W* to denote fitness and E(*W*) for expected fitness. We assumed a population of host plants in which individuals can follow one of two resistance strategies: (1) SAR, where resistance is affected by active responses which are stimulated by attempted infection and include systemic responses in unchallenged tissue; or (2) constitutive resistance (CON), in which resources are allocated to resistance irrespective of the probability of disease. Within the SAR type, priming occurs in response to environmental cues that indicate the likelihood of subsequent fitness-reducing pathogen attack and can result from both pathogenic and avirulent microbes. We assume here that the cue is an initial attempted infection, but the general model is compatible with a range of other potential stimuli. We tested the SAR strategy under two theoretical scenarios, one where SAR is complementary to CON resistance (i.e., plants may possess SAR in addition to CON, but all plants have CON) and one where SAR is an alternative to CON resistance (i.e., SAR evolves as a replacement for CON, and plants cannot possess both strategies). The hypotheses encapsulated within the game theory model are as follows:

1. We assume that the CON strategy is a universal baseline as there are no known plant species lacking constitutive resistance, and we assume that the strategy is inherently costly in terms of resource allocation and fitness in the absence of disease.
2. We assume that SAR priming is not 100% accurate, with the potential for both false positive errors (priming occurs but is not subsequently followed by pathogen attack) and false negative errors (pathogen attack occurs in the absence of a prior priming event).
3. For the situation in which SAR and CON are complementary strategies, CON is assumed to result in higher fitness than both unprimed SAR and primed SAR in the absence of disease, and primed SAR is assumed to result in lower fitness than unprimed SAR. In the presence of disease, we assume that primed SAR results in the highest fitness, followed by unprimed SAR, followed by CON.
4. For the situation where SAR and CON are alternative strategies, we consider two scenarios. In the absence of disease, we assume that CON results in lower fitness than unprimed SAR but higher fitness than primed SAR. When disease is present, the primed SAR strategy results in the highest fitness, followed by the unprimed SAR and finally the CON strategy.
5. Within any individual generation, disease is assumed to be a binary condition, so that fitness depends only on whether individuals are diseased or not.

### 4.3. Expected Fitness

We denote a fixed duration generation in which disease is absent as *D−* and one in which it occurs as *D+* and can rank the relative fitness provided by the SAR and CON strategies as follows:

*W*_CON|*D−*_ > *W*_SAR|*D−,−prime*_ > *W*_SAR|*D−,+prime*_ > *W*_SAR|*D+,+prime*_ > *W*_SAR|*D+,−prime*_ > *W*_CON|*D+*_

In the situation where the strategies are mutually exclusive the ranking is:

W_SAR|*D−,−prime*_ > *W*_CON|*D−*_ > *W*_SAR|*D−,+prime*_ > *W*_SAR|*D+,+prime*_ > *W*_SAR|*D+,−prime*_ > *W*_CON|*D+*_

Individuals with SAR can exist in one of two states prior to the initiation of disease, primed (*+prime*) or unprimed (*−prime*). For any individual plant, disease status during its lifetime is either *D+* or *D−*, and its relative fitness will be one of the six values listed above. However, expected fitness for a strategy over a number of generations will be the probability-weighted average of the fitnesses in the two environments (*D+* and *D−*), where the proportion of the total number of generations experiencing each type provides the weights. The probability that disease occurs, Pr(*D+*), is equal to *p*, and the probability that no disease occurs, Pr(*D−*), is equal to (1-*p*). The expected fitness, E(*W*), is a function of *p*. For the SAR strategy, an additional probability affects fitness, the probability of errors in the priming process. We denote the probability of false positive priming as FPP, and the probability of true negative priming is (1-FPP). Similarly, the probability of false negative priming is denoted FNP, with corresponding probability of (1-FNP) of a true positive priming event.

In a *D−* environment the expected fitness of SAR, E(*W*_SAR_), is [(FPP × *W*_SAR|*D−,+prime*_ + (1 − FPP)*W*_SAR|*D−,−prime*_]. Similarly, in a *D+* environment the E(*W*_SAR_) is [(1 − FNP)*W*_SAR|*D+,+prime*_ + FNP × *W*_SAR|*D+,−prime*_]. Over a sufficiently long period, the first of these fitness values will be achieved in a proportion of (1-*p*) years and the second in *p* years, and so the expected fitness is given by the probability-weighted sum of the two components, leading to an expression for E(*W*_SAR_) which is a linear function of *p*, as shown in Equation 1:

(1) $$E\left(W_{SAR}\right)=\left(1-p\right)\times \left[FPP\times W_{SAR|D-,+prime}+\left(1-FPP\right)\times W_{SAR|D-,-prime}\right]+p\times \left[\left(1-FNP\right)\times W_{SAR|D+,+prime}+FNP\times W_{SAR|D+,-prime}\right].$$

The corresponding case for E(*W*_CON_) is simplified by not having the terms for the priming error probabilities, as shown in Equation (2):

(2) $$E\left(W_{CON}\right)=\left(1-p\right)\times W_{CON|D-}+p\times W_{CON|D+}.$$

If E(*W*_SAR|*D−*_) > E(*W*_CON|*D−*_) but E(*W*_CON|*D+*_) > E(*W*_SAR|*D+*_), the solutions to Equations 1 and 2 will intersect at a particular value of *p*, p*, which represents the disease incidence at which the CON and SAR strategies have equal fitness. The value of p* is given by Equation 3:

(3) $$p^{*}=\frac{E\left(W_{CON|D-}\right)-E\left(W_{SAR|D-}\right)}{E\left(W_{SAR|D+}\right)-E\left(W_{SAR|D-}\right)-E\left(W_{CON|D+}\right)+E\left(W_{CON|D-}\right)}.$$

**Figure 5:**
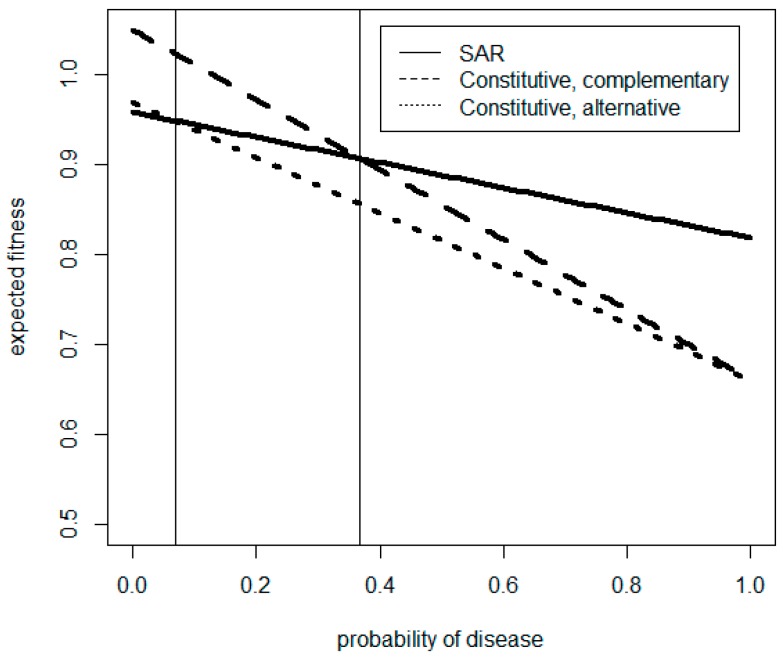
Solutions for Equations 1 and 2 corresponding to the values of relative fitness, E(*W*), from Figure 1. showing relative fitness payouts for populations with systemic acquired resistance (SAR) under a false positive rate of 40% and a false negative rate of 20% and/or constitutive resistance, where the disease incidence, p*, at which point strategies have equal expected fitness is marked by vertical solid lines at 0.368 for the complementary constitutive population and 0.069 for the alternative constitutive population.

The numerator on the right-hand side of Equation 3 is the difference in fitness between the two strategies when disease is absent, while the denominator is the difference between the rates of change in expected fitness of each strategy with increasing probability of disease. Note that if (*W*_SAR|*D−*_ > *W*_CON|*D−*_) and (*W*_SAR|*D+*_ > *W*_CON|*D+*_) (or if both of these inequalities are reversed) then the lines specified by Equations 1 and 2 will not intersect, and either SAR or CON (depending on the direction of the inequalities) will have higher expected fitness at all values of *p*. The solutions of Equations 1 and 2 corresponding to the values of E(*W*) in Figure 1 are shown in Figure 5, for illustration, with a false positive priming rate of 40% and a false negative priming rate of 20%.

The fitness values for the different plant disease resistance strategies in the *D−* and *D+* environments are defined as described in Equations 1 and 2. The game theory model defines the hypothetical winning and losing strategies in each generation, dependent on the numerical values of the parameters for each strategy and whether or not disease occurs. While the game theory model shows the deterministic outcome of the game in terms of expected fitness, it does not include any direct mechanism through which the differences in fitness might operate, nor does it demonstrate how stochastic effects arising from errors in the priming mechanism might lead to transient displacement of the plant population from its hypothetical long term stationary composition. In order to explore these effects, the game theory model for each generation is embedded in a stochastic simulation in which fitness is expressed through competition for reproductive success in a randomly varying environment with known long-term disease incidence.

### 4.4. Stochastic Simulation

The relative fitness values calculated from the synoptic analysis were incorporated into a game theory-based model using the R statistical programming language [38]. The model was designed so that each simulation run represented 1000 fixed duration generations of the hypothetical plant populations. In addition to the four relative fitness parameters calculated from the synoptic analysis, fitness parameters for a plant population with only constitutive (CON) disease resistance were estimated for healthy and diseased states. The assumptions about the different fitness values for the SAR and CON strategies are provided in the game theory description (above). Total population size was maintained at 1000 individuals, and both populations were projected over time, with the SAR population starting at five individuals and the constitutive population starting at 995 individuals. The small proportion of the population made up of the SAR group represents a new evolutionary lineage attempting to invade the resident type (constitutive-only resistance). In one scenario the new strategy had both SAR and constitutive resistance, in the other it had SAR instead of constitutive resistance. 

The probability for a disease epidemic to occur in any given time step (generation) was defined prior to each simulation experiment. The presence or absence of an epidemic is determined in each time step using a Bernoulli random variable with this predefined mean proportion. Parameters are also included for the chances of false negatives and false positives occurring in the SAR population; these relate to the accuracy of the plant population’s SAR priming and determine the relative fitness of the SAR phenotype in conjunction with disease incidence and relative fitness values in the presence and absence of disease. The accuracy of the priming system is determined in each generation of the simulation. At each time step, a Bernoulli random variable is generated to denote whether it is a D− or D+ environment in the current generation. Independent of that choice, a value is selected from a random uniform distribution. If that value is greater than the false negative probability of the priming system and the Bernoulli variable indicates D+, the SAR population has “chosen” the correct strategy. Likewise, if the value is greater than the chance for a false positive and the environment is D−, the SAR population has also chosen correctly. If the randomly generated value is less than or equal to the predefined chances for false negatives or positives, the SAR population has chosen incorrectly.

### 4.5. Translating Relative Fitness into Reproduction

At each time point, the new SAR and CON population sizes are determined according to the following equations, respectively:

(4) $$SAR_{t+1}=\frac{SF_{t+1}\times SAR_{t}}{SF_{t+1}\times SAR_{t}+CF_{t+1}\times CON_{t}}\times 1000,$$

(5) $$CON_{t}=1000-SAR_{t},$$

where *SAR* is the size of the SAR population, *CON* is the size of the constitutive population, *SF* is the fitness value for the SAR population, and *CF* is the fitness value for the constitutive population. These equations are used in each time step until one of the population sizes drops to zero, at which point, if it occurs, the simulation stops.

### 4.6. Numerical Simulation

A numerical simulation using the game theory model was developed to determine pathogen pressure necessary for individuals using the SAR strategy to have higher fitness than those using constitutive defenses alone. Scenarios assessed for both complementary and alternative strategy scenarios included five levels of disease certainty and five levels of FPP and FNP. Disease certainty was tested at 10%, 20%, 30%, 40%, and 50% for the scenario where SAR and CON are complementary strategies. Disease certainty was tested at 1%, 5%, 10%, 20%, and 30% for the scenario where SAR and CON are alternative strategies. Disease certainty levels were selected based on preliminary model results for the two scenarios. Probabilities for false positives and negatives were tested as follows: 1) FNP = 0%, FPP = 0% (perfect efficiency); 2) FNP = 10%, FPP = 20%; 3) FNP = 20%, FPP = 40%; 4) FNP = 30%, FPP = 60%; and 5) FNP = 40%, FPP = 80% (least efficient). The rate for a false positive was arbitrarily set at always twice that of the rate for a false negative under the assumption that unnecessary allocation of resources to priming is less costly than suffering pathogen infection. Each parameter value combination of disease incidence and error rate was tested in the simulation for at least 1000 generations using 100 replicates. The 100 replicate runs were averaged for information on overall trends within the model. For parameter value combinations where the populations had not stabilized after 1000 generations, the model was run for an additional 5000–20,000 generations, as needed to achieve stabilization.

### 4.7. Using the Model

The R package “plyr” is required to run this model. Specific instructions for using the model are embedded within the R Code (Supplementary Materials). Lines beginning with a “#” are either comments that are not used as code to run the model or unnecessary information that is “commented out” when running the model under different parameters. Disease probability and probability for a false positive may be set as desired and are both set to a default value of 0.4. The complementary strategy scenario is the default with fitness value for the constitutive population in the absence of disease set to 1.05; this line of code should be commented out when running the model under the alternative scenario (e.g., #TN = 1.05) and the “#” should be removed from the following line of code (e.g., TN = 0.97) to change the fitness value for the constitutive population. Placing the legend for the SAR fitness analysis graph that is produced at the end of the model requires clicking on the desired location within the graph window.

## Figures and Tables

**Table 1:** Meta-analysis of fitness costs or benefits of salicylic acid-mediated induced resistance to chemical or biological inducers in various plant species in presence or absence of pathogen pressure.

Reference	Inducing		Variable	Additional	n	Fitness Values		
Number	Agent		Measured	Treatments		UP,D ^z^	P,H ^y^	P,D ^x^
26	*Fusarium circinatum*		Growth	None	4	1.20	1.01	0.96
27	BION		Seed yield	No nutrients	10	-	0.98	-
				Med. nutrients	10	-	0.73	-
				High nutrients	10	-	0.59	-
28	*Perenospora hyoscyami*		Growth	None	30	-	1.39	-
31	BION		Seed yield	Low N	7	-	0.95	-
				Low-med N	7	-	0.72	-
				Med-high N	7	-	0.10	-
				High N	7	-	0.45	-
30	*Blumeria graminis*		Seed yield	None	15	0.74	0.93	-
29	Saccharin		Seed yield	None	10	0.84	0.78	1.05
32	BABA		Seed yield	10 mg/L BABA	10	0.62	1.02	0.96
				25 mg/L BABA	10	0.62	1.11	1.00
				60 mg/L BABA	10	0.62	0.69	0.67
	BTH		Seed yield	None	10	0.62	0.63	0.51
							
Scaled averages:					157	0.711	0.896	0.845
±SE						±0.018	±0.026	±0.028

^z^ where UP,D represents unprimed diseased plants as a proportion of the unprimed healthy control; ^y^ where P,H represents primed healthy plants as a proportion of the unprimed healthy control; ^x^ where P,D represents primed diseased plants as a proportion of the unprimed healthy control.

**Table 2:** Meta-analysis of fitness costs or benefits of salicylic acid-mediated induced resistance to chemical or biological inducers in various plant species in presence or absence of pathogen pressure. Simulation dynamics and final population size for hypothetical plant population with systemic acquired resistance (SAR) after competition with hypothetical population without SAR for at least 1000 generations where constitutive alone is more fit in the absence of disease; averages are from 100 runs of a game theory model based on a meta-analysis on fitness costs of SAR.

Disease	FPP ^y^	FNP ^x^	Transition ^w^		Plateau ^v^		Transition ^w^
Certainty ^z^			Generation	SE	Generation	SE	Population
10%	0%	0%	-	-	344	0.0	0
	20%	10%	-	-	97	0.0	0
	40%	20%	-	-	60	0.0	0
	60%	30%	-	-	42	0.0	0
	80%	40%	-	-	30	0.0	0
20%	0%	0%	526	34.5	3846	33.8	870
	20%	10%	-	-	590	0.0	0
	40%	20%	-	-	229	0.0	0
	60%	30%	-	-	87	0.0	0
	80%	40%	-	-	67	0.0	0
30%	0%	0%	140	29.9	478	0.4	1000
	20%	10%	290	34.0	941	14.1	980
	40%	20%	-	-	16335	0.0	0
	60%	30%	-	-	448	0.0	0
	80%	40%	-	-	240	0.0	0
40%	0%	0%	78	24.5	469	0.0	1000
	20%	10%	113	29.9	668	0.0	1000
	40%	20%	199	35.2	716	17.1	970
	60%	30%	1035	47.4	4318	47.6	660
	80%	40%	-	-	795	0.0	0
50%	0%	0%	54	23.3	150	0.1	1000
	20%	10%	69	27.0	218	0.3	1000
	40%	20%	94	30.4	271	0.2	1000
	60%	30%	137	32.2	445	10.0	990
	80%	40%	270	36.3	939	31.4	890

^z^ where disease certainty represents the probability for an epidemic to occur in any given generation; ^y^ where FPP represents the probability for a false positive epidemic prediction in any given generation; ^x^ where FNP represents the probability for a false negative epidemic prediction in any given generation; ^w^ where transition represents the average generation when the SAR population becomes >50% of the total population; listed with associated population standard error; ^v^ where plateau represents the average generation when the SAR population stabilizes; listed with associated population standard error.

**Table 3:** Simulation dynamics and final population size for hypothetical plant populations with systemic acquired resistance (SAR) after competition with hypothetical population without SAR for at least 1000 generations where constitutive resistance alone is less fit in absence of disease; averages are from 100 runs of a game theory model based on a meta-analysis on fitness costs of SAR.

Disease	FPP ^y^	FNP ^x^	Transition ^w^		Plateau ^v^		Final SAR
Certainty ^z^			Generation	SE	Generation	SE	Population
1%	0%	0%	164	6.4	399	0.0	1000
	20%	10%	505	22.5	1339	0.1	1000
	40%	20%	-	-	406	0.0	0
	60%	30%	-	-	81	0.0	0
	80%	40%	-	-	46	0.0	0
5%	0%	0%	130	11.8	319	0.0	1000
	20%	10%	276	21.3	749	0.1	1000
	40%	20%	-	-	3468	0.0	0
	60%	30%	-	-	132	0.0	0
	80%	40%	-	-	54	0.0	0
10%	0%	0%	104	14.8	258	0.1	1000
	20%	10%	182	22.9	472	0.1	1000
	40%	20%	631	32.7	3820	10.0	990
	60%	30%	-	-	467	0.0	0
	80%	40%	-	-	97	0.0	0
20%	0%	0%	75	16.7	187	0.1	1000
	20%	10%	105	21.4	269	0.1	1000
	40%	20%	177	26.1	592	0.4	1000
	60%	30%	493	32.2	1778	17.1	970
	80%	40%	-	-	717	0.0	0
30%	0%	0%	57	17.5	147	0.1	1000
	20%	10%	73	22.6	200	0.2	1000
	40%	20%	102	25.1	271	0.1	1000
	60%	30%	165	29.4	488	0.3	1000
	80%	40%	397	33.2	2091	19.7	960

^z^ where disease certainty represents the probability for an epidemic to occur in any given generation; ^y^ where FPP represents the probability for a false positive epidemic prediction in any given generation; ^x^ where FNP represents the probability for a false negative epidemic prediction in any given generation; ^w^ where transition represents the average generation when the SAR population becomes >50% of the total population; listed with associated population standard error; ^v^ where plateau represents the average generation when the SAR population stabilizes; listed with associated population standard error.

## Supplementary Materials

The following are available online at https://www.mdpi.com/2223-7747/8/7/219/s1, R Code.
